# Estimating the number of clusters via a corrected clustering instability

**DOI:** 10.1007/s00180-020-00981-5

**Published:** 2020-05-18

**Authors:** Jonas M. B. Haslbeck, Dirk U. Wulff

**Affiliations:** 1 Psychological Methods Group, University of Amsterdam, Amsterdam, The Netherlands; 2 Center for Cognitive and Decision Science, University of Basel, Basel, Switzerland; 3 Center for Adaptive Rationality, Max Planck Institute for Human Development, Berlin, Germany

**Keywords:** Cluster analysis, k-means, Stability, Resampling

## Abstract

We improve instability-based methods for the selection of the number of clusters *k* in cluster analysis by developing a corrected clustering distance that corrects for the unwanted influence of the distribution of cluster sizes on cluster instability. We show that our corrected instability measure outperforms current instability-based measures across the whole sequence of possible *k*, overcoming limitations of current insability-based methods for large *k*. We also compare, for the first time, model-based and model-free approaches to determining cluster-instability and find their performance to be comparable. We make our method available in the R-package cstab.

## Introduction

A central problem in cluster analysis is selecting the number of clusters *k*. This problem is typically approached by assuming the existence of a true number of clusters $k^*$ that can be estimated via an objective function that defines the quality of a clustering. Different definitions have been proposed and it is generally accepted that the usefulness of a definition depends on the clustering problem at hand (see e.g., Friedman et al. 2001; Hennig 2015).

Most definitions characterize the quality of a clustering in terms of a distance metric that depends on the locations and cluster assignments of the clustered objects. Methods relying on such definitions select *k* by trading-off the magnitude of the distance metric or some transformation of it against the magnitude of *k*. The most commonly used distance metric is the within-cluster dissimilarity *W*(*k*) of within-cluster object pairs averaged across all clusters. When selecting *k* based on this metric it is assumed that *W*(*k*) exhibits a kink at the true cluster number $k = k^*$. This is because adding more clusters beyond $k^*$ will decrease *W*(*k*) only by a relatively small amount, since new clusters are created from clusters that already are relatively homogeneous. All methods focusing on the distances between objects and clusters, in one way or another, aim to identify this kink. Two examples are the Gap statistic (Tibshirani et al. 2001) and the Jump statistic (Sugar and James 2003). Related metrics are the Silhouette statistic (Rousseeuw 1987), which is an index of cluster separation rather than variance, and a variant thereof, the Slope statistic (Fujita et al. 2014).

In contrast, the approach investigated in this paper defines a ‘good clustering’ in terms of its instability in response to perturbations of the data. Accordingly, instability-based methods select *k* to be the value that minimizes the instability of the clustering. Instability-based methods are attractive because they are not based on a specific metric for the distance between objects and have been shown to perform at least as well as state-of-the-art distance-based methods (e.g., Ben-Hur et al. 2001; Tibshirani and Walther 2005; Hennig 2007; Wang 2010; Fang and Wang 2012).

In this article, we show that the results of two the existing instability-based approaches, the *model-based* approach (Fang and Wang 2012) and the *model-free* approach (Ben-Hur et al. 2001), depend on the distribution of cluster sizes *M*. As a result, both approaches produce biased estimates of ${k}^*$, especially when the list of candidate *k* is not restricted to small numbers. To address this problem, we develop a corrected cluster instability measure that corrects for the influence of *M*. We show that our corrected instability measure outperforms current instability measures across the whole sequence of possible *k*. We also compare, for the first time, model-based and model-free approaches to determine cluster-instability and find that their performance is comparable. We make our method available in the R-package cstab, which is available on The Comprehensive R Archive Network (CRAN).

## Clustering instability

Let $\mathbf {X} = \{X_1, \dots , X_n \} \in \mathbb {R}^{n \times p}$ be *n* samples from an unknown distribution $\mathcal {P}$ defined on $\mathbb {R}^p$. We define a clustering $\psi : \mathbb {R}^{p} \mapsto \{1, \dots , K\}$ as a mapping from objects $X_i \in \mathbb {R}^p$ to $k \in {\{1,\ldots ,K\}}$ clusters where the clustering $\psi$ is learned from data by clustering algorithm $\Psi (\mathbf {X}, k)$.

A clustering $\psi$ is considered stable if it is robust against perturbations of the data. Specifically, under a stable clustering, two objects $X_1, X_2$ that occupy the same cluster in a clustering $\psi _a$ based on the original data $\mathbf {X}$ tend to also occupy the same cluster in a clustering $\psi _b$ based on a perturbed data $\widetilde{\mathbf {X}}$ and vice versa for objects not occupying the same cluster. This notion of pair-wise agreement and disagreement between two clusterings is the basis for defining a clustering distance and, in turn, the measure of *clustering instability* (e.g., Ben-David et al. 2006; Fang and Wang 2012)

### Definition 1

(*Object-pair Disagreement*) The pairwise disagreement of any pair of clusterings $\psi _a(\cdot )$ and $\psi _b(\cdot )$ for a fixed pair of objects $X_1$ and $X_2$ is defined as

$$\begin{aligned} a(\psi _a(X_1), \psi _b(X_2)) = | \mathbb {I}_{\{ \psi _a(X_1) = \psi _a(X_2) \}} - \mathbb {I}_{\{ \psi _b(X_1) = \psi _b(X_2) \}} |, \end{aligned}$$

where $\mathbb {I}_{(E)}$ is the indicator function for the event *E*.

**Fig. 1:**
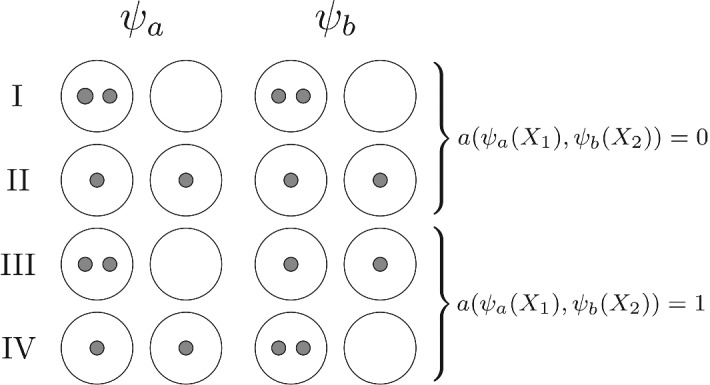
Schematic illustration of the four possible clustering distance configurations for the clusterings $\psi _a, \psi _b$ and two objects $X_1, X_2$, for the simplest non-trivial examples with two clusters

Figure 1 displays the four possible configurations that can occur in the indicator functions in Definition 1 for the example of two clusters and two objects $X_1, X_2$: if $\psi _a, \psi _b$ agree on whether any two objects occupy the same cluster or not (I and II), then $a(\psi _a(X_1), \psi _b(X_2)) = 0$. Conversely, if $\psi _a, \psi _b$ disagree (III and IV), then $a(\psi _a(X_1), \psi _b(X_2)) =1$.

Using the above definition, we define clustering distance as the expected disagreement $a(\psi _a(X_i), \psi _b(X_j))$ over all possible pairs (*i*, *j*), given probability distribution $\mathcal {P}$.

### Definition 2

(*Clustering Distance*) The distance between a pair of clusterings $\psi _a$ and $\psi _b$ is defined as

$$\begin{aligned} d(\psi _a, \psi _b) = \mathbb {E}_{X_i,X_j \sim P} \; a(\psi _a(X_i), \psi _b(X_j)), \end{aligned}$$

where the expectation is taken with respect to the probability distribution $\mathcal {P}$.

Now, using the above definition, we define clustering instability as the expected clustering distance for pairs of clusterings obtained for repeated perturbations of the data.

### Definition 3

(*Clustering instability*) The clustering instability of clustering algorithm $\Psi (X, k )$ is defined as

$$\begin{aligned} s(\Psi , k) = \mathbb {E}_{\widetilde{\mathbf {X}}_a, \widetilde{\mathbf {X}}_b \sim \mathcal {P}^n} \left[ d (\psi _a, \psi _b) \right] , \end{aligned}$$

where the clusterings $\psi _a$ and $\psi _b$ are obtained from two independent samples $\widetilde{\mathbf {X}}_a$ and $\widetilde{\mathbf {X}}_b$ with *n* observations drawn from $\mathcal {P}$, and the expectation is taken with respect to $\mathcal {P}$.

Since $s(\Psi , k)$ is the expectation of $d(\psi _a, \psi _b) \in [0,1]$ over pairs of independent samples from $\mathcal {P}$, it also takes values in [0, 1]. Given the definition of clustering instability, we estimate the true number of clusters $k^*$ by choosing the value of $k \in \{1, \dots , K\}$ that minimizes clustering instability:

1 $$\begin{aligned} \hat{k} = \arg \min _{2 \le k \le K} s(\Psi , \mathbf {X}, k). \end{aligned}$$

In the following two sections we describe two approaches to compute $s(\Psi , k)$ for a given data set $\mathbf {X}$ and clustering algorithm $\Psi$. The two approaches differ with respect to which pairs of objects are used to determine the clustering distance.

## Model-based clustering instability

The first approach computes clustering instability based on all objects contained in the data set, which requires that objects not contained in perturbations of the data set must also be assigned to a cluster. This can only be achieved using a clustering algorithm $\Psi (\cdot , k )$ that models the entire object space $\mathbb {R}^p$ as a partition into *k* non-empty subsets. An example clustering algorithm meeting this requirement is the k-means algorithm, which partitions $\mathbb {R}^p$ into *k* Voronoi cells (Hartigan 1975).

To calculate clustering instability using the model-based approach, we must, further, address that the definitions of clustering instability (Definition 3) and clustering distance (Definition 2) imply that independent samples are drawn from $\mathcal {P}$ and that the distance is calculated for all objects in $\mathcal {P}$ although $\mathcal {P}$ is unknown. Following Fang and Wang (2012), we address this problem by using the non-parametric bootstrap. That is, we repeatedly draw samples with replacement from $\mathbf {X}$ instead of $\mathcal {P}$ and approximate the expectation in Definition 3 by averaging over the finite number of bootstrap sample pairs. We could also have used a cross-validation (CV) scheme (Tibshirani and Walther 2005; Wang 2010); however, the non-parametric bootstrap (Fang and Wang 2012) has been found for perform better than CV (Fang and Wang 2012). The model-based algorithm then addresses the initial problem by determining the cluster assignment for all objects in *X* with respect to the clusterings of two bootstrap samples, before computing the clustering distance based on all pairs $X_i, X_j \in X$ utilizing the full partitioning of $\mathbb {R}^p$.
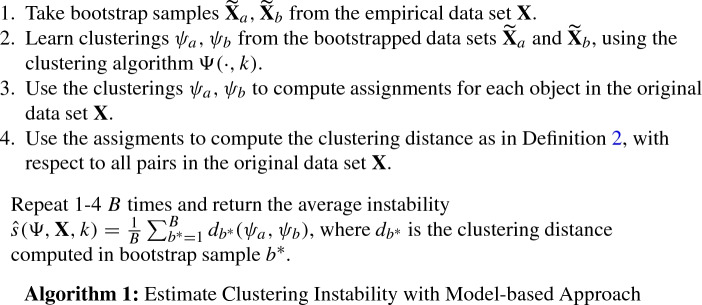


The model-based approach can be used with all clustering algorithms that fully partition $\mathbb {R}^p$, including spectral clustering (Ng et al. 2002) as described in Bengio et al. (2003). However, the model-based approach is not compatible with certain popular algorithms such as, for instance, hierarchical clustering (Friedman et al. 2001), which do not learn a full partitioning of $\mathbb {R}^p$ as required by step 3 of Algorithm 1. This shortcoming can be addressed by using an additional classifier (e.g., *k* nearest neighbors) to assign clusters to unseen objects. However, a simpler alternative, sidestepping this issue, exists in the model-free approach described in the following section.

## Model-free clustering instability

The model-free approach (Ben-Hur et al. 2001) sidesteps the requirement of a full partitioning of $R^p$ by computing the clustering distance in Definition 2 with respect to all pairs (*i*, *j*) of unique objects contained in *both* $\widetilde{\mathbf {X}}_{a}$ and $\widetilde{\mathbf {X}}_{b}$. As a result, no assignments of new objects to clusters are necessary, and therefore any clustering algorithm can be used.
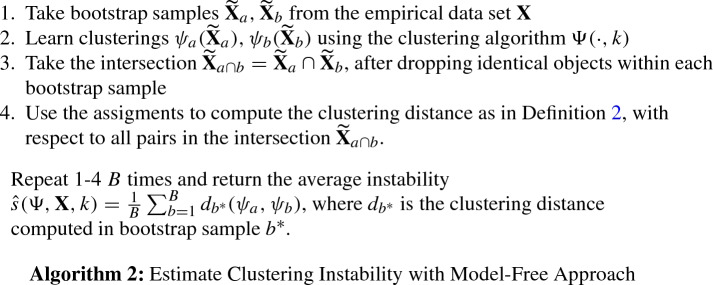


A potential cost of this flexibility is that Algorithm 2 compared to Algorithm 1 computes clustering instability only on approximately 40 % of the original data^1^, suggesting that a larger number of pairs of bootstrap samples *B* has to be sampled to achieve the same reliability as the model-based approach.

## A corrected clustering instability

No matter which of the two approaches one chooses, the desired behavior of clustering instability $s(\Psi , k)$ is to return small values for *k*s close to a theoretical $k^*$, and the smallest value for $k=k^*$. However, for the instability-based approaches described above, this is not generally the case. We will illustrate that clustering instability $s(\Psi , \mathbf {X}, k)$ heavily depends on the distribution of cluster sizes $M \in \{m_1, \dots , m_k \}$ implied by a clustering $\psi$ for $\mathbf {X}$ and by extension on the candidate *k*. This dependency introduces both noise and bias into the estimation of *k*.

The following examples illustrate the problem. Consider the clustering distance in Definition 2. The expectation can only be nonzero if it is possible for at least one pair $X_i, X_j$to be in the same cluster in one clustering and in different clusters in the other. This can only occur for $1<k<n$, as for $k=1$ the two objects $X_i, X_j$ would be forced into the same cluster and for $k=n$ they are forced into their own clusters (clusters are per definition nonempty). For the remaining range of $1<k<n$, the distance can become larger than 0. However, for values close to *n*, clustering distance will remain close to zero, because disagreement across clustering is only possible for a few pairs. For example, for $k=n-1$, the two clusterings can disagree only with respect to a single pair. On the other hand, $k=2$ allows for a much larger distance, since the clustering algorithm can in principle disagree with respect to all pairs. Note that the exact relationship of $d(\psi _a, \psi _b)$ and k, of course, also depends on the clustering algorithm and the data generating distribution $\mathcal {P}$.

To make above mentioned dependencies explicit, we rewrite Definition 2 in the following equivalent form:

2 $$\begin{aligned} {\begin{matrix} d(\psi _a, \psi _b) = \mathbb {E} \Big [ &{}\; \mathbb {I}_{\{ \psi _a(X_i) = \psi _a(X_j) \}} \times \mathbb {I}_{\{ \psi _b(X_i) \ne \psi _b(X_j) \}} \\ + &{}\; \mathbb {I}_{\{ \psi _a(X_i) \ne \psi _a(X_j) \}} \times \mathbb {I}_{\{ \psi _b(X_i) = \psi _b(X_j) \}} \Big ] . \end{matrix}} \end{aligned}$$

Using the identity $E[XY] = E[X]E[Y] + \text {cov}(X, Y)$, the definition of correlation $\text {cor}(X, Y) = \text {cov}(X, Y) / (\sqrt{\text {var}(X)} \sqrt{\text {var}(Y)})$ for random variables *X*, *Y*, and the shorthand $\mathbb {I}_{s} = \mathbb {I}_{ \psi _s(X_i) = \psi _s(X_j) }$ for $s \in \{a, b\}$, the equation in Definition 2 can be rearranged in the following way:

3 $$\begin{aligned} {\begin{matrix} d(\psi _a, \psi _b) &{} = \mathbb {E} (\mathbb {I}_a ) \times \mathbb {E} (1-\mathbb {I}_b)\\ &{}\quad +\, \mathbb {E} (1-\mathbb {I}_a) \times \mathbb {E} (\mathbb {I}_b )\\ &{}\quad + \, \text {cor} (\mathbb {I}_a, 1-\mathbb {I}_b) \times \sqrt{\text {var}(\mathbb {I}_a)} \times \sqrt{\text {var}(1-\mathbb {I}_b)}\\ &{}\quad + \, \text {cor} (1-\mathbb {I}_a,\mathbb {I}_b) \times \sqrt{\text {var}(1-\mathbb {I}_a)} \times \sqrt{\text {var}(\mathbb {I}_b)} . \end{matrix}} \end{aligned}$$

This representation of $d(\psi _a, \psi _b)$ shows that only the terms $\text {cor} (\mathbb {I}_a, 1-\mathbb {I}_b)$ and $\text {cor} (1-\mathbb {I}_a, \mathbb {I}_b)$ actually capture how well clusterings agree across bootstrap samples. All other terms, i.e., the expectations $\mathbb {E} (\mathbb {I}_s)$ and variances $\text {var} (\mathbb {I}_s)$, only concern the individual clusterings by themselves independent of the respective other clusterings. Crucially, however, these additional terms also depend on *k* via the distribution of cluster sizes *M*, producing the unwanted dependencies outlined above.

In order to remove these influences from $d(\psi _a, \psi _b)$ we next derive expressions for $\mathbb {E} (\mathbb {I}_s)$ and $\text {var} (\mathbb {I}_s)$, under the simplifying assumption that the probability of each event $\psi _s(X_i) = \psi _s(X_j)$, with $s \in \{a, b\}$, is constant for all pairs $(X_i, X_j) \sim P$. The assumption renders $\psi _s(X_i) = \psi _s(X_j)$ a Bernoulli event, allowing us to estimate $\mathbb {E} (\mathbb {I}_s)$ using

4 $$\begin{aligned} \mathbb {E} (\mathbb {I}_s) = \frac{\sum _{1 \le i \le k} {{m^i_s}\atopwithdelims (){2}}}{{{n}\atopwithdelims (){2}}} , \end{aligned}$$

where $m^i_s \in M_s = \{m^1_s, \ldots , m^k_s\}$ is the sizes of cluster *i* in clustering $\psi _s$. That is, we determine $\mathbb {E} (\mathbb {I}_s)$ by summing across clusters the number of possible object pairs in each cluster and then normalize by the total number of pairs given *n*. Using the above assumption, we can, furthermore, estimate $\text {var} (\mathbb {I}_s)$ using

5 $$\begin{aligned} \text {var} (\mathbb {I}_s) = \text {var} (1-\mathbb {I}_s) = \mathbb {E} (\mathbb {I}_s) (1- \mathbb {E} (\mathbb {I}_s)) . \end{aligned}$$

With these results and the shorthands $c_1 =\mathbb {E} (\mathbb {I}_a ) \mathbb {E} (1-\mathbb {I}_b) + \mathbb {E} (1-\mathbb {I}_a) \mathbb {E} (\mathbb {I}_b )$, $c_2 = \sqrt{\text {var}(\mathbb {I}_a)} \times \sqrt{\text {var}(1-\mathbb {I}_b)} = \sqrt{\text {var}(1-\mathbb {I}_a)} \times \sqrt{\text {var}(\mathbb {I}_b)}$, and the fact that $\text {cor} (\mathbb {I}_a, 1-\mathbb {I}_b) = \text {cor} (1-\mathbb {I}_a, \mathbb {I}_b)$, we define our corrected clustering distance as

6 $$\begin{aligned} d^c(\psi _a, \psi _b) = .5 \times \frac{d(\psi _a, \psi _b) - c_1}{c_2} = \text {cor} (\mathbb {I}_a, 1-\mathbb {I}_b). \end{aligned}$$

Using the above result, we define our corrected clustering instability measure $s^c(\Psi , k)$ consistent with Definition 3 as the expected corrected clustering distance. To the extent that our simplifying assumption is valid, the corrected clustering distance would no longer depend on *M* and capture only the disagreement between clusterings. Of course, in reality, we must expect the probability of two pairs sharing a cluster to vary across pairs, implying that our correction is likely imperfect. Nonetheless, if $c_1$ and $c_2$ substantially influence clustering distance, then we can expect the use of our corrected clustering instability to improve the performance of existing, uncorrected instability-based methods.

**Fig. 2:**
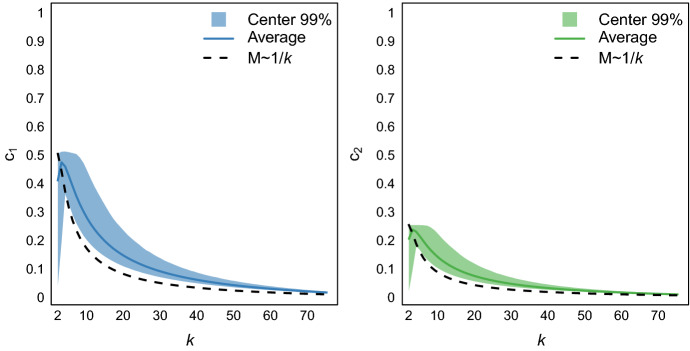
Simulated $c_1$ and $c_2$ across $k \in \{1, \dots , 75 \}$ for randomly generated *M* and $n=100$ objects. The shaded areas in the background show the center 99% of values due to variation in *M* for a given *k*. The solid lines represent the average for *M* drawn randomly (solid) and $m_1, \ldots , m_k = 100/k$ (dashed)

To illustrate the impact of $c_1$ and $c_2$ on clustering distance, we simulated $c_1$ and $c_2$ on the basis of cluster sizes $M_1, M_2 \sim Multinomial(\Pi )$ with $\Pi \sim Dirichlet(\mathbf {1})$ being the unknown distribution of cluster sizes in *P* and assuming $n=100$. Figure 2 shows the average $c_1$ (left panel) and $c_2$ (right panel) and their ranges (center 99%) across $k \in \{2, 3, \dots , 50\}$. We see that both $c_1$ and $c_2$ vary dramatically within and across *k*. Overall, both $c_1$ and $c_2$ are maximal for small values of *k*, with maximums for $k=3$ when drawn according to $\Pi$, and quickly taper off approaching 0 as *k* grows large. Being positively related to $c_1$ and $c_2$, the uncorrected clustering distance will therefore always be small for large *k*, irrespective of the location of $k^*$.

**Fig. 3:**
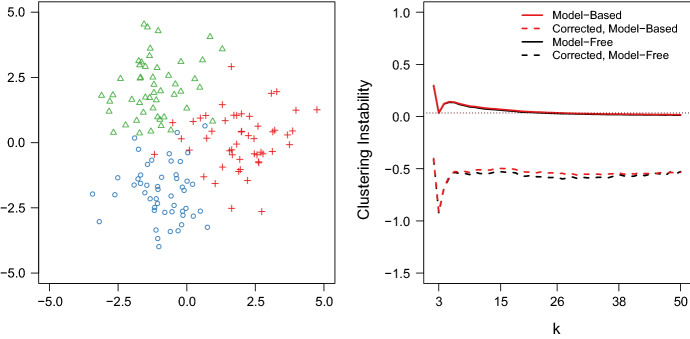
Left: mixture of three (each n = 50) 2-dimensional Gaussians with zero covariance and $\sigma _i = 1$. Right: instability path for the model-based (red) and model-free instability approach (black), both corrected (dashed) and uncorrected (solid). The horizontal lines indicate the local minimum of the instability path at $k^* = 3$ for each method. The estimate $\hat{k}$ will be incorrect (too large) if we consider *k*s with an instability below the corresponding horizontal line (color figure online)

The consequences for clustering instability are easily observed. Figure 3 shows the clustering instability obtained for the clustering problem studied by Fang and Wang (2012) using the corrected and uncorected clustering distance. As expected, the uncorrected clustering instability (solid line) tapers off as *k* is increasing with the consequence that for $k>25$ (model-based) and $k>23$ (model-free) the clustering instability becomes smaller than the value obtained for $k = k^* = 3$. The clustering instability using the corrected clustering distance, however, does not show this undesirable behavior. Instead, it shows a clearly defined global minimum at $k = k^*$ and no tapering off for larger *k*. The clustering instability using the corrected clustering distance therefore permits a more accurate estimation of $k^*$ across the entire range of *k*. As a result, it is no longer necessary to constrain the candidate set for *k* to small values to avoid $\hat{k} = \max k$ (cf., Fang and Wang 2012; Ben-Hur et al. 2001). In the next section, we use numerical experiments to demonstrate the performance of clustering instability using the corrected clustering distance to estimate $k^{*}$ across several realistic settings.

## Numerical experiments

We now turn to the numerical evaluation of the performance of uncorrected and corrected instability-based methods across four scenarios. This will include a comparison of the model-free and model-based instability-based approaches to the performance of four popular distance-based methods for estimating $k^*$.

### Data generation

**Fig. 4:**
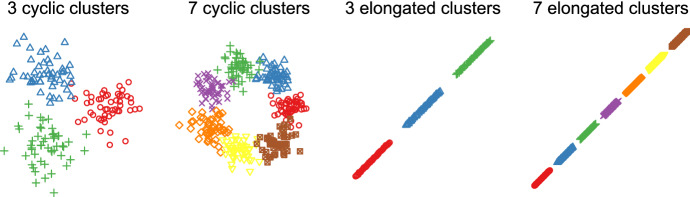
First column: three Gaussians with $\sigma = .1$ and $n=50$ placed on a circle; second column: seven Gaussians with $\sigma = .04$ and $n=50$ placed on a circle; third column: three elongated clusters in three dimensions (only the first two shown); fourth column: seven elongated clusters in three dimensions (only the first two shown)

We generated data from Gaussian mixtures as illustrated in Fig. 4. For the first scenario with $k^*=3$, we equally distributed the means of three Gaussians $(\sigma = 0.15)$ on a unit circle and sampled $n=50$ from each Gaussian. For the second scenario with $k^*=7$, we equally distributed the means of seven Gaussians $(\sigma = 0.04)$ on a unit circle and sampled $n=50$ from each Gaussian. The total sample sizes of the first and second problem are, thus, 150 and 350, respectively. The third and fourth scenario used elongated clusters similar to those in Tibshirani and Walther (2005): we generated $n = 50$ equally spaced points along the diagonal of a 3-dimensional cube with side length $[-5, 5]$, and added uncorrelated Gaussian noise ($\mu = 0$ and $\sigma _i = 0.1$) to each data point. We then replicated these data points to reflect the true number of clusters $k^* = 3$ and $k^* = 7$, for the third and fourth scenario, respectively, and placed them along a line separated by a distance of 15. As above, the total sample sizes of the third and fourth scenario were 150 and 350, respectively. We provide code to fully reproduce our simulation results in the Online Supplementary Material. Note that the illustration of the third and fourth scenario in Fig. 4 omits the third dimension.

### Comparison plan

The main goal of our numerical experiments is to compare our novel corrected clustering instability method to the standard, uncorrected clustering instability methods. However, to also learn about the relative merits of instability-based methods, we compare their performance to the performance of popular distance-based methods for *k*-selection. Note that these methods imply different definitions of a ’good’ clustering (see introduction). Thus, strictly speaking the different methods solve different problems. Nonetheless, in practice, all of these methods are applied for the same purpose. In some way, the various methods can be understood as different heuristic solutions to a given problem (here, the four scenarios described in Sect. 6.1).

We consider the following four distance-based methods: the Gap Statistic (Tibshirani et al. 2001), the Jump statistic (Sugar and James 2003), the Slope statistic (Fujita et al. 2014), and a Gaussian mixture model. The Gap statistic simulates uniform data of the same dimensionality as the original data and then compares the gap between the logarithm of the within-cluster dissimilarity *W*(*k*) for the simulated and original data. It selects the value of *k* for which this gap is largest. The Jump statistic computes the differences of the within-cluster distortion at *k* and $k-1$ (after transformation via a negative power) to select the value of *k* that produced the largest differences in distortions. The Slope statistic is based on the Silhouette statistic *Si*(), and selects *k* to maximize $[Si(k) - Si(k-1) ] Si(k)^v$, where *v* is a tuning parameter. Finally, the Gaussian mixture model selects *k* as the number of components in the mixture model yielding the lowest Bayesian Information Criterion (BIC) (Schwarz 1978). We used the BIC as a model selection criterion, since it has been shown to be a consistent estimator for the number of components (clusters) in finite Gaussian mixture models (Leroux 1992), and because it has been shown to outperform other information criteria in simulations (Steele and Raftery 2010).

We evaluated the *k*-selection methods using the k-means clustering algorithm (Hartigan 1975). The k-means algorithm was restarted 10 times with random starting centroids in order to avoid local minima. Dick et al. (2014) showed that 10 restarts for k-means were sufficient for two clustering problems that match the problems considered here. For all methods, we considered the sequence $k = \{2, 3, \dots , 50\}$. For the instability-based methods, we used 100 pairs of bootstrap samples (see Algorithm 1 and  2). To maximize comparability, we used the same set of random seeds across the instability-based methods (within the same iteration).

**Table 1:** Estimated number of clusters in four different scenarios for 100 iterations

	3 circular clusters, 2 dimensions																		
	2	**3**	4	5	6	7	8	9	10	11	12	13	14	15	16	17	18	19	20+
Model-based	0	**68**	0	0	0	0	0	0	0	0	0	0	0	0	0	0	0	0	32
Model-based (C)	0	**100**	0	0	0	0	0	0	0	0	0	0	0	0	0	0	0	0	0
Model-free	0	**43**	0	0	0	0	0	0	0	0	0	0	0	0	0	0	0	0	57
Model-free (C)	0	**100**	0	0	0	0	0	0	0	0	0	0	0	0	0	0	0	0	0
Gap statistic	0	**100**	0	0	0	0	0	0	0	0	0	0	0	0	0	0	0	0	0
Jump statistic	0	**0**	0	0	0	0	0	0	0	0	0	0	0	0	0	0	0	0	100
Slope statistic	0	**96**	4	0	1	0	0	0	0	0	0	0	0	0	0	0	0	0	0
Gaussian mixture	0	**100**	0	0	0	0	0	0	0	0	0	0	0	0	0	0	0	0	0

	7 circular clusters, 2 dimensions																		
	2	3	4	5	6	**7**	8	9	10	11	12	13	14	15	16	17	18	19	20+
Model-based	0	0	0	0	0	**0**	0	0	0	0	0	0	0	0	0	0	0	0	100
Model-based (C)	0	0	0	0	0	**87**	13	0	0	0	0	0	0	0	0	0	0	0	0
Model-free	0	0	0	0	0	**0**	0	0	0	0	0	0	0	0	0	0	0	0	100
Model-free (C)	0	0	0	0	0	**91**	9	0	0	0	0	0	0	0	0	0	0	0	0
Gap statistic	0	0	0	0	0	**100**	0	0	0	0	0	0	0	0	0	0	0	0	0
Jump statistic	0	0	0	0	0	**8**	0	0	0	0	0	0	0	0	0	0	0	0	92
Slope statistic	0	0	0	0	0	**31**	17	18	18	9	5	1	0	0	1	0	0	0	0
Gaussian mixture	0	0	0	0	0	**99**	1	0	0	0	0	0	0	0	0	0	0	0	0

	3 elongated clusters, 2 dimensions																		
	2	**3**	4	5	6	7	8	9	10	11	12	13	14	15	16	17	18	19	20+
Model-based	0	**100**	0	0	0	0	0	0	0	0	0	0	0	0	0	0	0	0	0
Model-based (C)	0	**100**	0	0	0	0	0	0	0	0	0	0	0	0	0	0	0	0	0
Model-free	0	**100**	0	0	0	0	0	0	0	0	0	0	0	0	0	0	0	0	0
Model-free (C)	0	**100**	0	0	0	0	0	0	0	0	0	0	0	0	0	0	0	0	0
Gap statistic	0	**0**	0	0	0	0	0	0	0	0	0	0	0	0	0	0	0	0	100
Jump statistic	0	**0**	0	0	0	0	0	0	0	0	0	0	0	0	0	0	0	0	100
Slope statistic	0	**100**	0	0	0	0	0	0	0	0	0	0	0	0	0	0	0	0	0
Gaussian mixture	0	**99**	0	0	1	0	0	0	0	0	0	0	0	0	0	0	0	0	0

	7 elongated clusters, 2 dimensions																		
	2	3	4	5	6	**7**	8	9	10	11	12	13	14	15	16	17	18	19	20+
Model-based	0	0	0	0	0	**0**	0	0	0	0	0	0	0	0	0	0	0	0	100
Model-based (C)	19	0	0	0	0	**42**	39	1	1	0	0	0	0	0	0	0	0	0	0
Model-free	0	0	0	0	0	**0**	0	0	0	0	0	0	0	0	0	0	0	0	100
Model-free (C)	19	0	0	0	0	**51**	30	0	0	0	0	0	0	0	0	0	0	0	0
Gap statistic	0	0	0	0	0	**0**	0	0	0	0	0	0	0	0	0	0	0	0	100
Jump statistic	0	0	0	0	0	**0**	0	0	0	0	0	0	0	0	0	0	0	0	100
Slope statistic	0	0	0	0	0	**68**	7	10	6	2	4	3	0	0	0	0	0	0	0
Gaussian mixture	0	0	0	1	0	**99**	0	0	0	0	0	0	0	0	0	0	0	0	0

### Results

Table 1 shows the estimated $\hat{k}$ over 100 iterations for each of the four scenarios and eight methods. Estimated $\hat{k} \ge 20$ are collapsed in the category ’20+’. We first focus on the results of the instability-based methods. For the first scenario with $k^*=3$ circular clusters, the uncorrected instability-based methods perform poorly, with about half of the estimates being correct, and the other half being in the category ’20+’. This poor performance was expected given the unfavorable behavior illustrated in Figs. 2 and 3. The corrected instability methods, however, mitigate this problem and accordingly show high performance. The pattern of results in the scenario with $k^* = 7$ is similar, only more pronounced. With the clustering problem being more difficult, uncorrected instability-based methods fail to identify $k^*$ in every iteration, whereas the corrected instability-based methods still successfully identify $k^*$ in the vast majority of cases. In the third scenario with $k^*=3$ elongated clusters, all instability-based methods show maximum performance. The favorable performance of all instability-based methods is due to the fact that the tail of the instability path for uncorrected methods did not undercut the local minimum at $k = 3$ for $k \ge 50$. In the fourth scenario with $k^*=7$ elongated clusters, the performance of the uncorrected methods drops to zero, whereas corrected instability methods are still able to identify $k^*$ in a considerable number of cases. Overall the results show that the corrected instability-methods perform better than the uncorrected ones.

We now turn to the performance of distance-based methods. The clear winner among this class of methods is the Gaussian mixture, which performs extremely well in all scenarios. This is what one would expect, since data was generated from a Gaussian mixture. Next, the Slope statistic performs reasonably well; however, the performance is much lower for $k^*=7$ than for $k^*=3$. The Gap statistic shows maximal performance for the circular clusterings, but drops to zero in for the elongated clusters. Finally, the Jump statistic shows poor performance in all scenarios. The reason for the bad performance of the Jump statistic is that its variance increases with increasing *k*. See Appendix B for a detailed illustration of this problem.

**Fig. 5:**
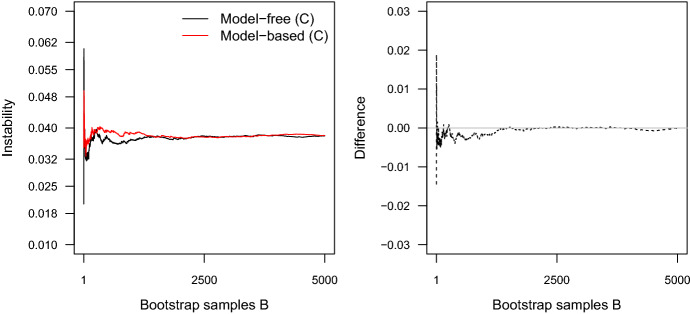
Left: The average instability for fixed $k=3$ up to bootstrap sample *b* for both the model-based (red) and model-free (black) instability approach. The data are those from Fig. 3. Right: The difference between the two functions (color figure online)

Our comparison revealed that corrected instability-based methods compare favorably to existing distance-based methods. With our proposed correction, instability-based methods outperform every distance-based method, except for the Gaussian mixture methods. However, without our proposed correction, it is almost always better to use any of the distance-based methods. For additional comparisons between the methods, consult Appendix A where we study small variations of the first and second scenario including additional noise dimensions.

Another noteworthy finding of our analysis is the near-equivalent performance of the model-based and the model-free instability approaches (see Table 1). To analyze whether the two methods converge for large *B*, we ran both methods using the scenario of Fig. 3 over a increasing number of $B \in \{1, 2, \dots , 5000\}$ pairs of bootstrap samples. Figure 5 shows that although both methods seem to stabilize in a small region around 0.038 they still show considerable variance even with 5000 bootstrap samples. It is thus unclear whether the two methods converge; however, they may converge for larger *B*. Furthermore, we evaluated the correlations between the instability paths of both approaches for the simulation reported in Table 1. They are between 0.98 and 1, suggesting that the two methods behave very similarly.

## Conclusions

We have proposed a correction for cluster-instability methods for estimating $k^*$, the true number of clusters in a dataset, and demonstrated that it enables accurate estimation of $k^*$ across the entire range of *k* by controlling for the unwanted influences of the distribution of cluster sizes *M*. We also have shown that instability-based methods, especially when using the proposed correction, can outperform established distance-based *k*-selection methods. Finally, we have compared model-based and model-free variants of the instability-based method and found them to be similar, but not identical. Together, these results corroborate the usefulness of cluster instability as an approach for estimating the number of clusters in a dataset.

Future research should extend our work in the following two ways. First, given the divergence of the model-based and model-free approaches, future research should study in closer detail the relative performance of the two across different situations. Second, future research should investigate more appropriate corrections by relaxing our simplifying assumption of equal probability for two objects occupying in the same cluster. That is, while our numerical experiments demonstrate the usefulness of using $d^c$, there is potential for more complex, better corrections.

## Figures and Tables

**Fig. 6:**
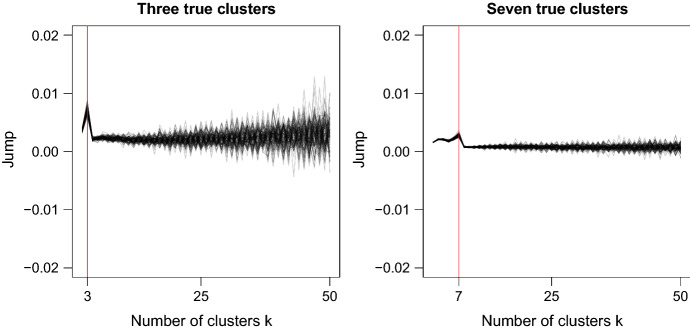
The Jump statistics along the sequence of considered *k* for each of the 100 simulation iterations. Left: for the scenario with three true clusters in two dimensions. Right: for the scenario with seven true clusters in two dimensions

## Appendix A: Additional numerical experiments

Table 2 shows the results of two additional scenarios that are adapted from scenarios one and two in Sect. 6.3 by adding eight dimensions of uncorrelated Gaussian noise with standard deviations $\sigma = 0.15$ in scenario 1 and $\sigma = 0.04$ in scenario 2.

**Table 2:** Estimated number of clusters in different scenarios

	3 circular clusters, 10 dimensions																		
	2	**3**	4	5	6	7	8	9	10	11	12	13	14	15	16	17	18	19	20+
Model-based	0	**49**	0	0	0	0	0	0	0	0	0	0	0	0	0	0	0	0	51
Model-based (C)	0	**100**	0	0	0	0	0	0	0	0	0	0	0	0	0	0	0	0	0
Model-free	0	**4**	0	0	0	0	0	0	0	0	0	0	0	0	0	0	0	0	96
Model-free (C)	0	**100**	0	0	0	0	0	0	0	0	0	0	0	0	0	0	0	0	0
Gap statistic	0	**100**	0	0	0	0	0	0	0	0	0	0	0	0	0	0	0	0	0
Jump statistic	0	**0**	0	0	0	0	0	0	0	0	0	0	0	0	0	0	0	0	100
Slope statistic	0	**97**	3	0	0	0	0	0	0	0	0	0	0	0	0	0	0	0	0
Gaussian mixture	0	**100**	0	0	0	0	0	0	0	0	0	0	0	0	0	0	0	0	0

	7 circular clusters, 10 dimensions																		
	2	3	4	5	6	**7**	8	9	10	11	12	13	14	15	16	17	18	19	20+
Model-based	0	0	0	0	0	**0**	0	0	0	0	0	0	0	0	0	0	0	0	100
Model-based (C)	0	0	0	0	0	**68**	32	0	0	0	0	0	0	0	0	0	0	0	0
Model-free	0	0	0	0	0	**0**	0	0	0	0	0	0	0	0	0	0	0	0	100
Model-free (C)	0	0	0	0	0	**83**	17	0	0	0	0	0	0	0	0	0	0	0	0
Gap statistic	0	0	0	0	0	**100**	0	0	0	0	0	0	0	0	0	0	0	0	0
Jump statistic	0	0	0	0	0	**0**	0	0	0	0	0	0	0	0	0	0	0	0	100
Slope statistic	0	9	91	0	0	**0**	0	0	0	0	0	0	0	0	0	0	0	0	0
Gaussian mixture	0	0	0	0	0	**100**	0	0	0	0	0	0	0	0	0	0	0	0	0

The performance is qualitatively similar to the performance reported in the main text. However, performance dropped for all methods as a result of the added noise, which rendered the clustering problem more difficult.

## Appendix B: Path of jump statistic

One reason for the bad performance of the Jump statistic is that the variance of the Jump size increases as *k* increases. We illustrate this problematic behavior of the Jump statistic in Fig. 6 using 100 iterations of scenario 1 (three circular clusters) and 2 (seven circular clusters) from the main text.

The figure plots the Jump statistic for each of the 100 iterations across $k \in \{2, 3, \dots , 10\}$. We see that the variance of the Jump statistic clearly increases for larger *k*. This implies that similarly to the uncorrected stability-based methods, the Jump statistic can only identify $k^*$, when the range of possible *k* is restricted to a small range around the true $k^*$.

## Footnotes

1 For large *n*, we have $P(a_j \in B_1 \wedge a_j \in B_2) = P(a_j \in B_1) P(a_j \in B_2) = (1-\frac{1}{e})^2.$
